# Multi-Target Screening and Experimental Validation of Natural Products from *Selaginella* Plants against Alzheimer's Disease

**DOI:** 10.3389/fphar.2017.00539

**Published:** 2017-08-25

**Authors:** Yin-Hua Deng, Ning-Ning Wang, Zhen-Xing Zou, Lin Zhang, Kang-Ping Xu, Alex F. Chen, Dong-Sheng Cao, Gui-Shan Tan

**Affiliations:** ^1^Xiangya School of Pharmaceutical Sciences, Central South University Changsha, China; ^2^Pharmacy Department, Xiangya Hospital, Central South University Changsha, China; ^3^College of Food Science and Technology, Central South University of Forestry and Technology Changsha, China; ^4^Center for Vascular Disease and Translational Medicine, Third Xiangya Hospital, Central South University Changsha, China

**Keywords:** Alzheimer, *Selaginella* plants, multi-target screening, multi-target SAR, BACE1, MAO-B

## Abstract

Alzheimer's disease (AD) is a progressive and irreversible neurodegenerative disorder which is considered to be the most common cause of dementia. It has a greater impact not only on the learning and memory disturbances but also on social and economy. Currently, there are mainly single-target drugs for AD treatment but the complexity and multiple etiologies of AD make them difficult to obtain desirable therapeutic effects. Therefore, the choice of multi-target drugs will be a potential effective strategy inAD treatment. To find multi-target active ingredients for AD treatment from *Selaginella* plants, we firstly explored the behaviors effects on AD mice of total extracts (TE) from *Selaginella doederleinii* on by Morris water maze test and found that TE has a remarkable improvement on learning and memory function for AD mice. And then, multi-target SAR models associated with AD-related proteins were built based on Random Forest (RF) and different descriptors to preliminarily screen potential active ingredients from *Selaginella*. Considering the prediction outputs and the quantity of existing compounds in our laboratory, 13 compounds were chosen to carry out the *in vitro* enzyme inhibitory experiments and 4 compounds with BACE1/MAO-B dual inhibitory activity were determined. Finally, the molecular docking was applied to verify the prediction results and enzyme inhibitory experiments. Based on these study and validation processes, we explored a new strategy to improve the efficiency of active ingredients screening based on trace amount of natural product and numbers of targets and found some multi-target compounds with biological activity for the development of novel drugs for AD treatment.

## Introduction

Alzheimer's disease (AD) is a progressive and irreversible neurodegenerative disorder which is considered to be the most common cause of dementia. With the acceleration of aging process in human society, AD prevalence is expected to reach the epidemic levels (Mount and Downton, 2006). Commonly, a majority of AD patients often have both of behavioral and psychological symptoms of dementia (BPSD). The behavioral characteristic includes the progressive loss of memory, the decline of cognitive function, the decrease of physical function and ultimately problems with communication, time and space disorientation and so on. The psychological symptom includes psychosis, depression, agitation and anxiety (Gauthier et al., 2010; Okura et al., 2011; Borisovskaya et al., 2014). Furthermore, the presence of BPSD usually exacerbates the morbidity and mortality associated with dementia. In more advanced stages, BPSD has a greater impact on social and economic than on the learning and memory disturbances and it has become the major impetus to force patients choosing primary home care and specialized psychogeriatric units. Unfortunately, the existing therapeutic approaches for BPSD are usually efficacy-limited and associated with serious adverse effects, such as the increasing risk of death (Cummings, 2000; U.S. Food and Drug Administration, 2005, 2008).

Although the molecular mechanism of AD pathogenesis has not been clearly understood, several hypotheses have been proposed for AD pathogenesis and their interconnections aggravate this disease a complex disorder (Šimić et al., 2017). The amyloid hypothesis (Goedert and Spillantini, 2006) is hallmarked by the neuropathological accumulation of amyloid beta (Aβ) plaques in the extracellular compartment and the intracellular accumulation of hyper-phosphorylated tau protein in the form of neurofibrillary tangles. The cholinergic hypothesis proposed a decreased level of acetylcholine in certain areas of brain (Craig et al., 2011). Oxidative stress hypothesis proposed the deregulation of endogenous detoxification redox systems and over-production of radical species leading to lipid peroxidation and nucleic acid mutations (Pratico, 2008). In addition, some other hypotheses, such as glutamatergic hypothesis (Bezprozvanny and Mattson, 2008), metal hypothesis (Bonda et al., 2011), and inflammatory hypothesis (Trepanier and Milgram, 2010) have also been proposed. Based on these pathogenesis, there are more than 200 enzymes or proteins related to AD, such as AchE, BACE1, GSK3β, MAO-B, GABA-A receptor, Glutamate receptor, and so on (Saura et al., 1994; Sathya et al., 2012; Fang et al., 2015; Yan et al., 2016). At present, licensed drugs approved for AD treatment are always based on single-target pharmacology. Now, there are two main categories of drugs for AD treatment: one is AchE inhibitor, including donepezil, rivastigmine, and galantamine. They can improve ACh level in the brain by decreasing the hydrolysis of ACh and are mainly used for mild to moderate AD treatment. The other one is N-methyl-D-aspartate antagonist (NMDA). The representative drug, memantine, is mainly used for the treatment of moderate to severe AD, but it is only licensed in several countries because of serious adverse drug reaction (Cummings, 2004; Standridge, 2004). Until now, the limitation of therapeutic treatments and their poor effectiveness make AD treatment become the current biggest medical problem in neurology. In fact, as described before, the complexity and multiple etiologies of AD make the single-target strategy difficult to obtain desirable therapeutic effects. Therefore, the choice of multi-target drugs will be a potential effective strategy in the treatment of AD and consequently the new chemical skeletons or active precursors with multi-target activities for AD therapy are inspired to be found.

As we all know, natural product is a highly valuable resource in searching for chemical precursors with potential bioactivity and few adverse effects because of their structural diversity. For example, biflavonoid glycosides from *Impatiens balsamina* show potential neuroprotective activity (Kim et al., 2017) and apigenin, quercetin show potent anti-Aβaggregation activity which is one of the major culprits in AD (Espargaró et al., 2017). Huperzine A (Hup A) is a highly selective, reversible and potent AChEI extracted from the Chinese medicinal herb *Huperzia serrata*. Compared with tacrine and donepezil, it has a higher bioavailability and potency but is less active toward BChE (Silva et al., 2014; Pisani et al., 2016). Nowadays, the purification of new chemical skeletons and activity screening from natural products still maintain sightless and accidental. Although more and more trace elements have been purified with the development of separation technology, it is still scarcely possible to carry out large-scale activity screening due to the contingency and trace outputs of separation. In recent years, with the rapid development of computer science and the accumulation of chemogenomics data, multi-target SAR model for active-ingredient screening was proposed as a useful method for seeking active compounds and target identification (Cao et al., 2012, 2014; He et al., 2013; Yao et al., 2016). As to the multi-target SAR, the SAR predictive model for each target protein is built based on the relationship between the chemical structure of active and inactive compound. This *in silico* method can give a preliminary screening and target identification for a large number of natural compounds with a prediction probability before the *in vitro* activity test is carried out.

Based on the previous researches, flavonoids show extensive pharmacological activities including anti-AD efficiency. In 2015, Duan SW has identified silibinin, a flavonoid, as a dual inhibitor of AChE and Aβ peptide aggregation for AD treatment (Duan et al., 2015). And then, Song X also proved that Silibinin can attenuate the inflammatory responses, increase glutathione (GSH) levels, decrease malondialdehyde (MDA) levels and upregulate autophagy levels in the Aβ_25−35_-injected rats (Song et al., 2017). What's more, Baicalein, *Scutellaria barbata* flavonoids, *Capparis spinose* flavonoids, and 4-dimethylamine flavonoid derivatives all show some degree of anti-AD activities in animal experiments or *in vitro* tests (Gu et al., 2016; Luo et al., 2016; Mohebali et al., 2016; Wu et al., 2016). Therefore, it's highly valuable and feasible to screen multi-target ingredients from flavonoids extracts for the treatment of AD.

In this study, we aimed to find multi-targets active ingredients for AD treatment from the flavonoids extracts of *Selaginella* plants. Firstly, we explored behavioral effects on AD mice of total extracts (TE) from *Selaginella doederleinii* by Morris water maze test. And then, we screened our home-database consisted of compounds extracted from *Selaginella* plants to hunt ingredients with anti-AD activity through multi-target SAR models *in silico*. Finally, the *in vitro* enzyme activity inhibitory test and the molecular docking experiment were applied to verify the prediction results and to find the potential active ingredients for the AD multi-targets treatment.

## Materials and methods

### Total extracts of *Selaginella* plants

Two hundred and fifty seven compounds were purified from *Selaginella* plants, including *Selaginella tamariscina, Selaginella pulvinata* Maxim, *Selaginella braunii* Baker, *Selaginella delicatula* (Desv.) Alston, *Selaginella moellendorfii* hieron, *Selaginella uncinate, Selaginella involven Spring, Selaginella doederleinii* Hieron. Total extracts (TE) were extracted using 75% ethanol and then freeze-dried into extractum. The suspensions of saline and freeze-dried extractum after ultrasonic vibration was orally administrated for AD mice.

### Morris water maze test

The learning and memory ability of AD mice were evaluated by Morris water maze test. Male specific-pathogen-free (SPF) grade male ICR mice (body weighing 18–22 g) were purchased from Hunan Provincial Experimental Animal Centers [Changsha, Hunan, China, Certificate No. SYXK (Xiang) 2012-0004] (Sun et al., 2009).

Mice were randomly divided into five groups (10 mice for each group), namely normal control group (NCG), model control group (MCG), low dose group (LDG, 50 mg/kg), middle dose group (MDG, 100 mg/kg) and high dose group (HDG, 200 mg/kg). To build the AD mice model, mice in MCG, LDG, MDG, and HDG were treated with D-gal (120 mg/kg, intraperitoneally) for 56 days (8 weeks), and the mice in normal group were treated with saline of the same volume for 56 days (8 weeks; dorsonuchal subcutaneous injection). After that, the TE suspensions of saline and freeze-dried extractum after ultrasonic vibration was orally administrated to the mice in LDG, MDG, and HDG for 42 days (6 weeks), and the mice in NCG and MCG were orally treated with saline of the same volume for 42 days (6 weeks). Finally, the spatial learning and memory ability of all the mice were tested by Morris water maze.

The equipment of Morris Water Maze were purchased from Anhui Zheng-hua biological equipment corporation and the test process followed to the relevant laboratory manual. Two indexes, the place navigation and spatial probe, were chosen as the main monitor elements to evaluate the spatial learning and memory ability of all the mice. The experimental method is divided into two parts: acquisition phase and probe trial. In the acquisition phase, we randomly put the head of the mouse into the wall of the pool and fix the starting position. After that, the time of finding the underwater platform was recorded. On the day after acquisition phase, the platform was removed and the probe trial began. The time of finding the position where the platform is located, the swimming distance and the number of crossing through the area where the platform is located were recorded as the spatial memory test indexes.

This study was carried out in accordance with the recommendations of “Laboratory Animals-Guideline of welfare and ethics, Ethics Committee of Hunan Provincial Experimental Animal Centers.” The protocol was approved by the “Ethics Committee of Hunan Provincial Experimental Animal Centers.”

### Multi-target SAR model and prediction of 257 compounds

According to previous studies published in recent years, we finally found 19 significant proteins related to AD (Cavalli et al., 2008; Fang et al., 2015). For these important AD-related proteins, we collected their ligands that are small, drug-like molecules from Binding database^1^. For each protein, activity data were filtered to keep only activity end-point points that have half-maximum inhibitory concentration (IC_50_), half-maximum effective concentration (EC_50_) or Ki values. A compound would be considered as a positive sample when its activity value was below 10 μM. Otherwise, this compound would be considered as a negative sample. Following this step, maybe some AD-related proteins have very little number of negative samples. To balance the number between positive samples and negative samples, we randomly selected certain number of compounds from other AD-related proteins to generate the negative samples for these AD-related proteins. The number of these selected negative samples together with inactive samples should be basically equal to the number of the active samples for these AD-related proteins. These prepared positive sets and negative sets were used for the subsequent model building. The detailed information of AD-related proteins and these datasets used for model building can be seen in Supporting Information (Supplementary Material).

For each protein, a series of high confidence SAR models were built by Random Forest (RF) and different fingerprint representations (FP2, MACCS, Daylight, ECFP2, ECFP4, and ECFP6). RF was introduced by Breiman and Cutler for regression and classification modeling in 2001 firstly (Breiman, 2001). The method is based upon an ensemble of decision trees, from which the prediction of a classification task is provided as the majority vote of the predictions of all trees. Recent studies have suggested that RF offers several striking features which make it very attractive for QSAR/QSPR studies. These include relatively high accuracy of prediction, built-in descriptor selection, and a method for assessing the importance of each descriptor to the model (Cao et al., 2011a,b; Yun et al., 2016). RFs were trained using the RF library in the statistical computing environment, R. All the fingerprints were calculated by some tools developed by our group: ChemDes, BioTriangle webserver and ChemoPy package (Cao et al., 2013; Dong et al., 2015, 2016). To improve the prediction ability of the SAR model, we assembled all fingerprint models to obtain the consensus models with average output. All the assembled models were validated by 5-fold cross validation and test set validation to demonstrate their prediction performance. In this part, some popular statistic parameters were applied to evaluate the performance of these classification models: true positive (TP); false negative (FN); true negative (TN); false positive (FP); sensitivity (SE); specificity (SP); accuracy (ACC); area under receiver operating characteristic curve (AUC). These classification evaluation parameters are defined as follows:

$$\begin{array}{l} \text{ SE}=\frac{\text{TP}}{\text{TP}+\text{FN}}\\ \text{ SP}=\frac{\text{TN}}{\text{TN}+\text{FP}}\\ \text{ACC}=\frac{\text{TP}+\text{TN}}{\text{TP}+\text{FP}+\text{TN}+\text{FN}} \end{array}$$

After a series of modeling and validation processes, we aimed to obtain reliable SAR models for above-mentioned AD-related proteins. And then, 257 compounds purified from *Selaginella* plants were predicted by these robust and practical models and their inhibitory activities were identified preliminarily for further study.

### Target enzyme inhibitory activity *in vitro*

For the compounds that have been regarded as active ingredients by the multi-target SAR models, the *in vitro* target enzyme inhibitory activity test was applied to verify their actual activity for AD treatment. The inhibitory activities were determined by fluorimetric method on Infinite M200 Multi scan Spectrum (Tecan, Swiss). Each concentration was analyzed in triplicate and IC_50_ values were determined by nonlinear regression of inhibition vs. log concentration plots, using GraphPad Prism 7 for Windows, Version 7.00 (GraphPad Software Inc.). BACE1 fluorescence resonance energy transfer assay kits were purchased from the Pan Vera Co and Monoamine Oxidase B (MAO-B) inhibitor screening kits were purchased from Bio Vision Inc.

In the BACE1 inhibition test, the assay was performed in 384-well plates. The assay solution was consisted of 10 μL test compounds (concentrations: 0.017, 0.050, 0.167, 0.500, 1.667, 5.000, 16.667, and 50.000 μM), 10 μL BACE1 substrate and 10 μL BACE1 enzyme. LY2811376 was selected as the reference compound with IC_50_ = 0.242 μM and the blank buffer was set as the negative control. The mixture was incubated for 60 min at room temperature. At the end, 10 μL BACE1 stop solution was added to stop the reaction and the fluorescence was detected at the Ex/Em = 545/585 (12 nm bandwidth) settings on Multi scan Spectrum.

In the MAO-B inhibition test, the assay was performed in 96-well plates. The assay solution was consisted of 10 μL test compounds(concentrations: 0.2, 0.4, 0.8, 1.6, 3.2, 6.4, 12.8, and 25.6 μM), 37 μL MAO-B assay buffer, 1 μL MAO-B substrate, 1 μL developer and 1 μL OxiRed Probe. Selegiline was used as the reference control with IC_50_ = 0.028 μM and the blank buffer was set as the negative control. The mixture was incubated for 10 min at 37°C. The fluorescence was measured at Ex/Em = 535/587 nm kinetically at 37°C for 10–40 min. Two points (T1 and T2) in the linear range of the plot were chosen and the corresponding fluorescence values (RFU1 and RFU2) were obtained to calculate the slope for all samples. The Calculation of % relative inhibition was following the manual of MAO-B inhibitor screening kit.

### Molecular docking simulation

To further verify the results of multi-target SAR prediction and enzyme inhibitory experiments, the molecular docking process was applied to simulate the binding position and binding affinity between the active compounds and target proteins. Generally speaking, docking is a computer simulation modeling technique used to predict the interaction between a ligand and a receptor active site, and is an important tool in structure-based drug design. The technique of docking is to position the ligand in different orientations and conformations within the binding site to calculate optimal binding geometries and energies. In this part, the molecular operating environment (MOE, version 2014.) was applied to carry out the molecular docking process. MOE's dock application searches for favorable binding modes between small- to medium-sized ligands and a not-too-flexible macromolecular target. For each ligand, a number of placements called poses are generated and scored. The score can be calculated as either a free energy of binding including among others solvation and entropy terms, or enthalpy based on polar interaction terms including metal ligation, or as qualitative shaped-based numerical value. According to the score values, ligands with different conformations can be ranked and the optimal structural conformation will be affirmed (Wang J. et al., 2015). To make the interactions with the binding site easy to see, the ligand interaction was carried out. It will automatically be loaded with a 2D diagram of the original ligand and a schematic representation of the binding site residues, with the important interactions between ligand and binding site shown. In this study, we selected two proteins as the docking acceptors: BACE1 (PDB ID: 1TQF) (Cobum et al., 2004); MAO-B (PDB ID: 2V5Z) (Binda et al., 2007). As a control, the original ligand included in the crystal structure should also be docked. A series of parameters were set: Dock: rescoring 1 = ASE; retain = 100; rescoring 2 = ASE; retain = 100. Configure force field: final gradient = 0.0001; maximum iterations = 1,000; force constant = 10; radius offset = 0.4. For rest parameters, the default treatment was applied.

## Results and discussion

### Behavioral evaluation of AD mice dealt with total extracts

**Figure 1:**
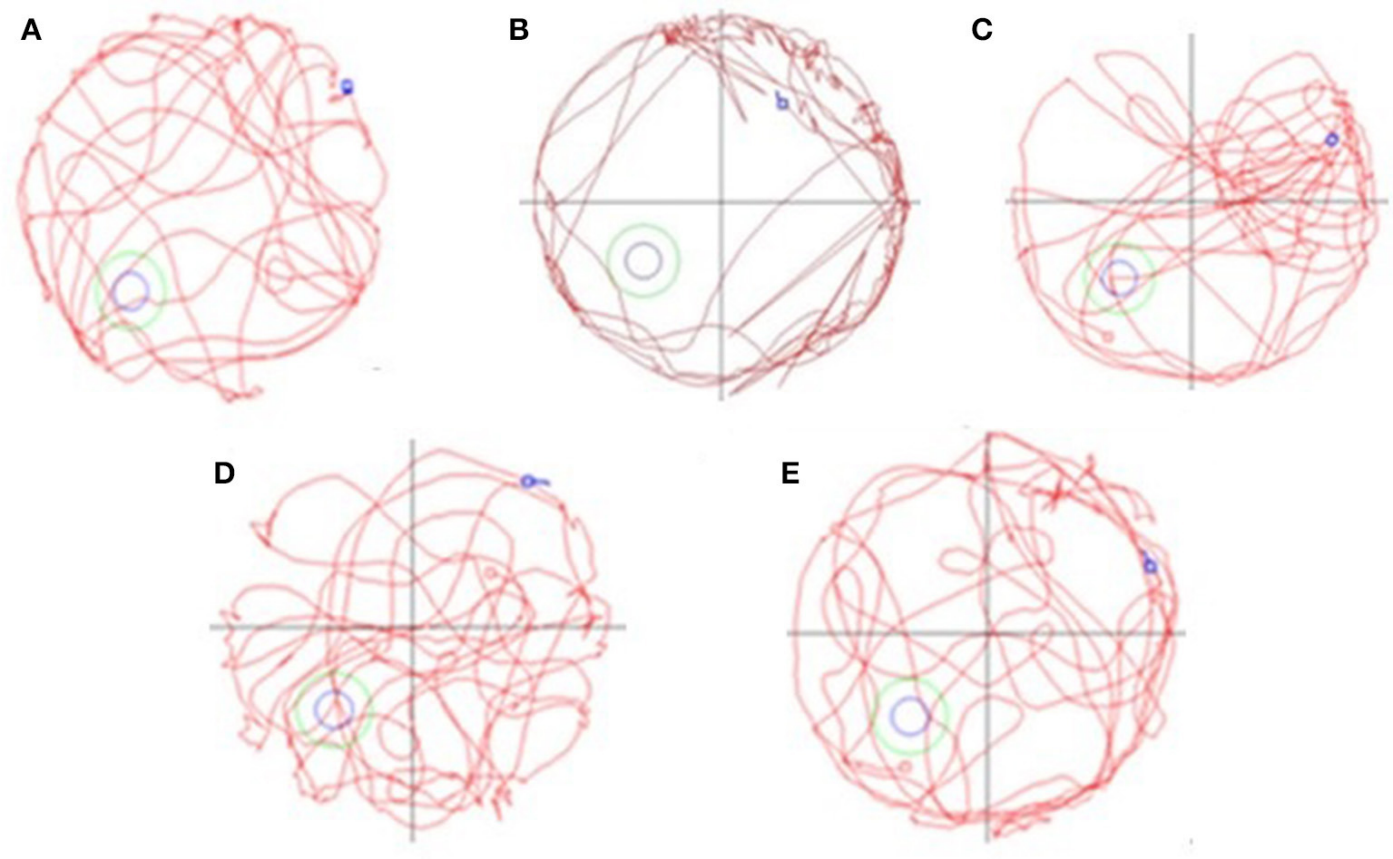
The spatial learning and memory ability of AD mice tested by Morris water maze [**(A)** NCG: normal control group; **(B)** MCG: model control group; **(C)** LDG: low dose group; **(D)** MDG: middle dose group; **(E)** HDG: high dose group]. This figure shows that TE has a remarkable improvement on learning and memory function for AD mice which mainly lies in the increased distance and this functional improvement is dose-dependent.

Learning and memory ability of AD mouse was evaluated by Morris water maze test in which the navigation and space exploration are used as indexes. There were five groups of mice under study and the behavioral results can be seen in Table 1 and Figure 1. In the Table 1, the residence time and residence distance of each quadrant, the total distance and the number through platform for each group of mice were listed. From the table, we can see that the residence time of MCG was significantly decreased (*P* < 0.05) in 1st and 4th quadrant compared with NCG. The stay intervals in 1st quadrant for MCG group were significantly lower than NCG (*P* < 0.05). However, the residence distance in 3rd quadrant increased prominently (*P* < 0.05). HDG showed longer distance in 1st quadrant (*P* < 0.05) and opposite trends in 3rd quadrant (*P* < 0.05) compared with MCG after 42 days' dosage. With respect to the total distance and numbers through platform, they were significantly reduced for MCG (*P* < 0.05) compared with NCG. They were increased significantly for HDG (*P* < 0.05) compared with MCG after 42 days' dosage. What's more, the crossing through number for MDG were also significantly increased (*P* < 0.05).

**Table 1:** The effects of TE on AD mice's behavior (*x* ± s, *n* = 10).

**Group**	**Dose (mg/kg)**	**1st quadrant**		**2nd quadrant**		**3rd quadrant**		**4th quadrant**		**Total distance**	**Speed**	**Crossing through number**
		**Time**	**Distance**	**Time**	**Distance**	**Time**	**Distance**	**Time**	**Distance**			
NCG	–	46.5 ± 6.2	906.1 ± 307.2	24.1 ± 6.5	741.5 ± 145.9	24.1 ± 6.5	419.0 ± 109.2	23.0 ± 4.3	544.5 ± 78.8	2778.4 ± 269.8	22.2 ± 0.8	4.0 ± 1.1
MCG	–	30.4 ± 2.3^*^	707.5 ± 122.0^*^	28.2 ± 1.8	750.4 ± 97.3	29.2 ± 1.8	580.7 ± 116.7^*^	18.9 ± 0.4^*^	421.1 ± 97.3	2459.9 ± 303.4^*^	20.5 ± 4.2	1.0 ± 0.0^*^
LDG	50	31.5 ± 5.4	833.1 ± 396.3	25.7 ± 3.4	685.7 ± 142.4	25.7 ± 4.0	706.9 ± 107.3	14.0 ± 0.1	723.6 ± 20.9	2420.7 ± 768.8	23.2 ± 5.6	1.5 ± 0.7
MDG	100	36.3 ± 1.9^+^	835.6 ± 295.4	30.4 ± 1.2	837.5 ± 49.8	30.4 ± 1.2	535.9 ± 110.2	29.8 ± 5.3^+^	349.3 ± 49.9	2659.7 ± 100.7	22.8 ± 1.6	2.0 ± 1.4^+^
HDG	200	39.9 ± 1.5^+^	854.6 ± 110.5^+^	35.8 ± 4.9	692.2 ± 262.2	16.8 ± 1.9^+^	453.5 ± 95.4^+^	29.3 ± 1.4^+^	468.8 ± 104.0	2729.4 ± 188.3^+^	20.2 ± 6.4	3.0 ± 1.4^+^

Compared with NCG

* P < 0.05, compared with MCG

+ *P < 0.05; Crossing through number, the number of crossing through the area where the platform is located; time, s; distance, cm; speed, cm/s*.

Figure 1 shows the navigation and space exploration for different groups of mice. From the figure, we can see that the spatial learning and memory ability of NCG were significantly increased, but there was an opposite trend for MCG and mice in MCG mainly ran along the cell wall. What's more, all the low, medium and high dose of TE can significantly increase the numbers of exploration platform. Considering both results in Table 1 and Figure 1, TE of *Selaginella* has a remarkable improvement on learning and memory function for AD mice. This result inspires us to further explore the effect of the chemical ingredients from *Selaginella* on AD treatment.

### Performance evaluation and the inhibitory activity prediction

Based on the results of the Morris water maze test, the TE of *Selaginella* plants show a potential benefit for AD treatment. To quickly screen the active ingredients from a number of compounds preliminarily, multi-target SAR models associated with AD-related proteins were constructed as described before. In this part, we finally obtained a series of ensemble predictive models for AD-related proteins. Their statistic results of 5-fold cross validation and test set validation were listed in Table 2. From this table, we can see that for each predictive model, the accuracy is good enough not only for cross validation (0.808–0.955) but also the test set validation (0.846–0.970). With respect to other statistic parameters, the similar results were obtained and it can be strong evidence for the good predictive ability of this model. Therefore, we have reasons to believe that these ensemble models are robust and practical and can be used to predict the inhibitory activity for a new compound in the early stage of drug discovery.

**Table 2:** The statistic results of these predictive models (5-fold cross validation and test set validation).

**Uniprot_ID**	**Gene name**	**5-fold cross validation**				**Test set validation**			
		**AUC**	**ACC**	**SE**	**SP**	**AUC**	**ACC**	**SE**	**SP**
P08908	HTR1A	0.950	0.904	0.884	0.924	0.961	0.921	0.911	0.931
Q9Y5N1	HRH3	0.985	0.955	0.944	0.967	0.991	0.970	0.967	0.973
P50406	HTR6	0.969	0.930	0.918	0.942	0.986	0.953	0.932	0.974
P22303	ACHE	0.892	0.845	0.818	0.871	0.944	0.903	0.886	0.922
Q99720	SIGMAR1	0.967	0.918	0.897	0.938	0.979	0.949	0.944	0.955
P11229	CHRM1	0.864	0.808	0.798	0.818	0.938	0.882	0.882	0.883
P49841	GSK3B	0.892	0.821	0.786	0.856	0.963	0.903	0.902	0.905
P06276	BCHE	0.879	0.820	0.797	0.842	0.926	0.855	0.862	0.848
P56817	BACE1	0.961	0.930	0.919	0.942	0.971	0.942	0.951	0.934
P27338	MAOB	0.888	0.826	0.809	0.842	0.941	0.877	0.860	0.895
P36544	CHRNA7	0.875	0.824	0.791	0.857	0.916	0.846	0.792	0.899
Q07343	PDE4B	0.957	0.914	0.884	0.945	0.974	0.942	0.953	0.930
P27815	PDE4A	0.951	0.917	0.911	0.924	0.962	0.922	0.910	0.934
Q13639	HTR4	0.963	0.943	0.921	0.965	0.976	0.950	0.920	0.977
P46098	HTR3A	0.894	0.853	0.810	0.896	0.928	0.892	0.833	0.949
Q96BI3	APH1B	0.942	0.899	0.917	0.882	0.973	0.948	0.963	0.935
P05067	APP	0.973	0.915	0.881	0.950	0.944	0.912	0.867	0.961
Q9NZ42	PSENEN	0.945	0.924	0.946	0.903	0.951	0.914	0.953	0.880
Q8WW43	APH1B	0.937	0.920	0.938	0.902	0.952	0.935	0.924	0.944

To evaluate the probability of inhibitory activity for the 19 AD-related targets, 257 compounds were purified from the TE including 143 flavonoids, 9 selaginellins and some other compounds. Before the inhibitory activity prediction by SAR models, the preliminary druggability evaluation was carried out to exclude some compounds that have no beneficial property for further drug development process. In this part, we mainly evaluated the molecular weight and two important ADME (absorption, distribution, metabolism, elimination) properties for druggability by corresponding QSAR models developed by our group: logD_7.4_ (the distribution coefficients at pH = 7.4) (Wang J. B. et al., 2015; Wang et al., 2017), and logPapp (the Caco-2 membrane permeability) (Wang et al., 2016). Based on previous studies, a good drug candidate should have a logD_7.4_ value smaller than 5, a logPapp value larger than −5.15 and a molecular weight smaller than 500. After excluding compounds that perform very poorly in at least two of three aforementioned properties, there were 238 compounds left for further activity screening.

As described before, the inhibitory activity of these 238 compounds were predicted by the multi-target SAR model. The predictive result for a new compound was outputted as a probability value. For each compound that was classified as active ingredient by SAR models, if its probability value >0.5, it is considered to be active, otherwise, it is inactive. From the predictive result, it can be seen that 54 flavonoids and 4 selaginellins present a good inhibitory correlation with MAO-B, 21 flavonoids may show BACE1 inhibitory activity. However, to improve the reliability of prediction, we apply the prediction probability of 0.8 as a cut-off value to select the active compounds for some related targets. As a result, 18 compounds with a probability value larger than 0.8 were extracted. These compounds were prepared for further validation in the inhibitory activity test and their detailed information can be seen in the Supporting Information (Supplementary Material).

### *In vitro* validation of inhibitory activity for target enzyme

Based on the prediction outputs, we focused on the screening of BACE1/MAO-B dual inhibitory activity of flavonoids and selaginellins. The enzyme BACE1 is considered as a prime target to design therapeutics for AD mainly because of that the catalysis process by BACE1 is the rate-limiting step in APP proteolysis and the BACE1 knock-out mice lacking Aβ production survives with normal physiology (Roberds et al., 2001). As the majorβ-secretase enzyme that initiates the generation of Aβ, BACE1 is undoubtedly a prime target for anti-Aβtherapy in AD (Ohno, 2016). The increase of MAO-B activity is associated with gliosis, which can result in higher levels of H_2_O_2_ and oxidative free radicals (Nebbioso et al., 2012). Thus, the MAO-B inhibitors are potential candidates for anti-AD drugs due to their capacity to regulate neurotransmitters and inhibit oxidative damage in the central nervous system.

**Figure 2:**
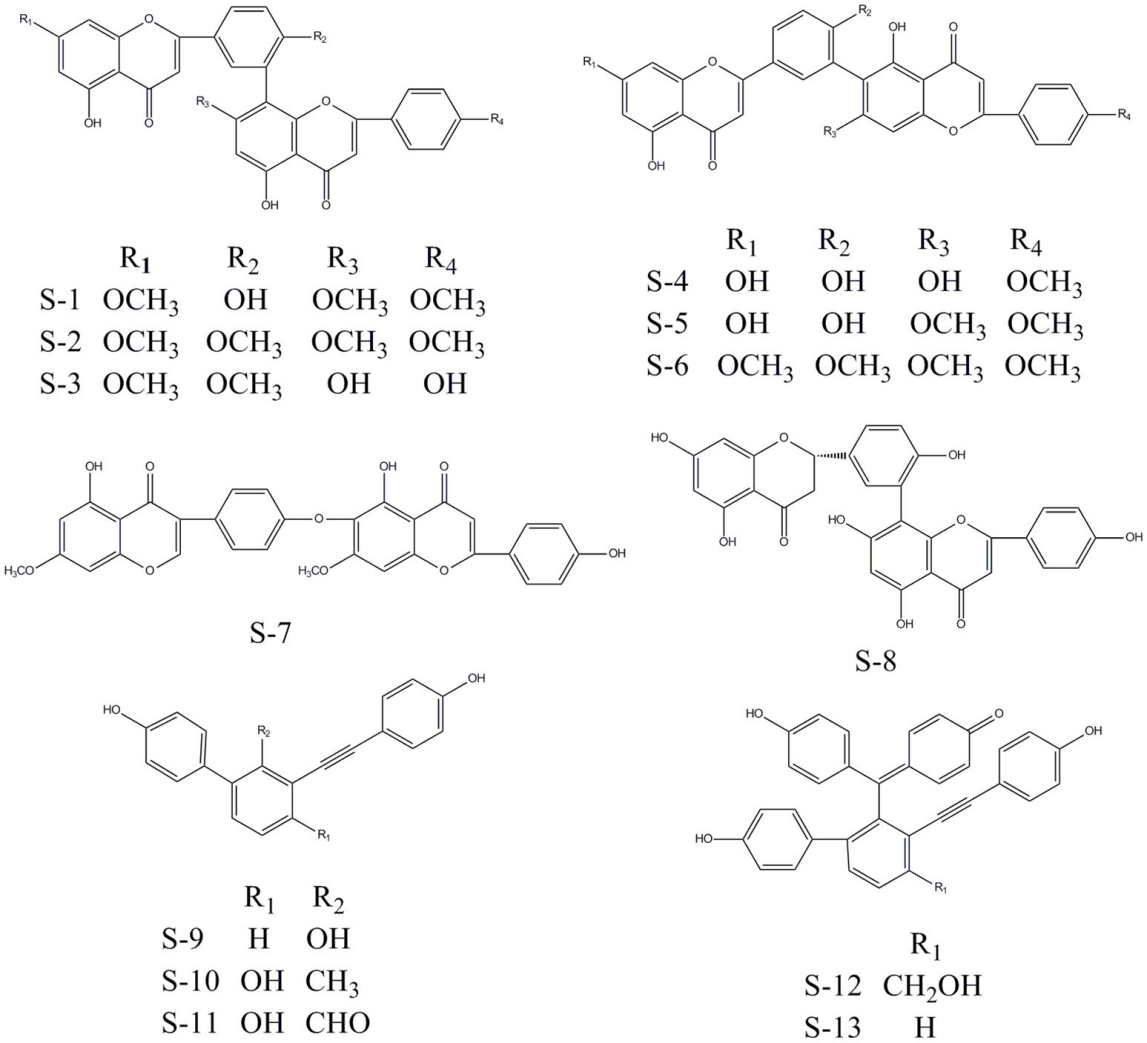
The chemical structures of 13 compounds that with inhibitory activity after multi-target SAR model prediction. Among them, eight are biflavones and the left five are selaginellins.

**Figure 3:**
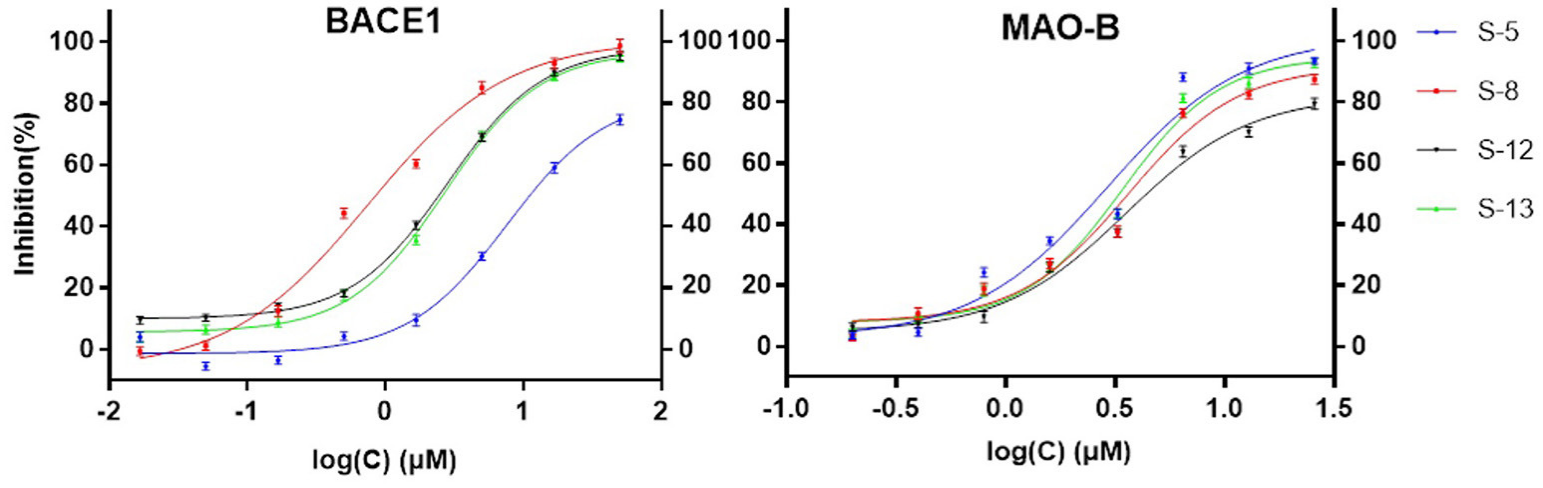
Verification of BACE1 and MAO-B Inhibition. This figure shows that all these four compounds (S-5, S-8, S-12, S-13) have good inhibitory activity in the *in vitro* validation test.

Considering the quantity of existing compounds in our laboratory, 13 compounds were chosen to carry out the inhibitory activity validation experiments. Their chemical structures were displayed in Figure 2 and their IC_50_ values can be seen in Table 3. To evaluate the inhibitory activity of these compounds, a threshold value of IC_50_ = 10 μM was applied. If a compound has a IC_50_ values smaller than 10 μM, it would be considered to be active. Otherwise, it is inactive. We can find that nine of them show good inhibition on BACE1 with IC_50_ values ranged from 0.7454 to 7.578 μM and five of them show good inhibition on MAO-B with IC_50_ values ranged from 2.913 to 8.813 μM. Among them, S-8, S-5, S-13, and S-12 all have significant dual BACE1/MAO-B inhibitory activities with IC_50_ values in the micromole magnitude and S-8 has been proved to be the most potent against BACE1 and MAO-B with IC_50_ values of 0.7454 and 3.619 μM, respectively. Among them, S-5 and S-8 are biflavones, S-12 and S-13 are selaginellins. The inhibitory curves for these four compounds were summarized in Figure 3.

**Table 3:** The IC_50_ values of 13 compounds under study.

**Compound**		**S-1**	**S-2**	**S-3**	**S-4**	**S-5**	**S-6**	**S-7**	**S-8**	**S-9**	**S-10**	**S-11**	**S-12**	**S-13**	**Z-factor**
IC_50_ (μM)	BACE1	70.89	17.20	2.75	81.93	7.58	20.70	3.97	0.75	4.32	3.40	2.27	2.82	2.72	0.93
	MAO-B	–^a^	15.74	11.72	13.89	2.91	8.81	23.17	3.62	18.21	10.24	–^a^	3.52	3.42	0.89

a *The IC_50_ value cannot be calculated in the predetermined concentration range*.

### Molecular docking

**Figure 4:**
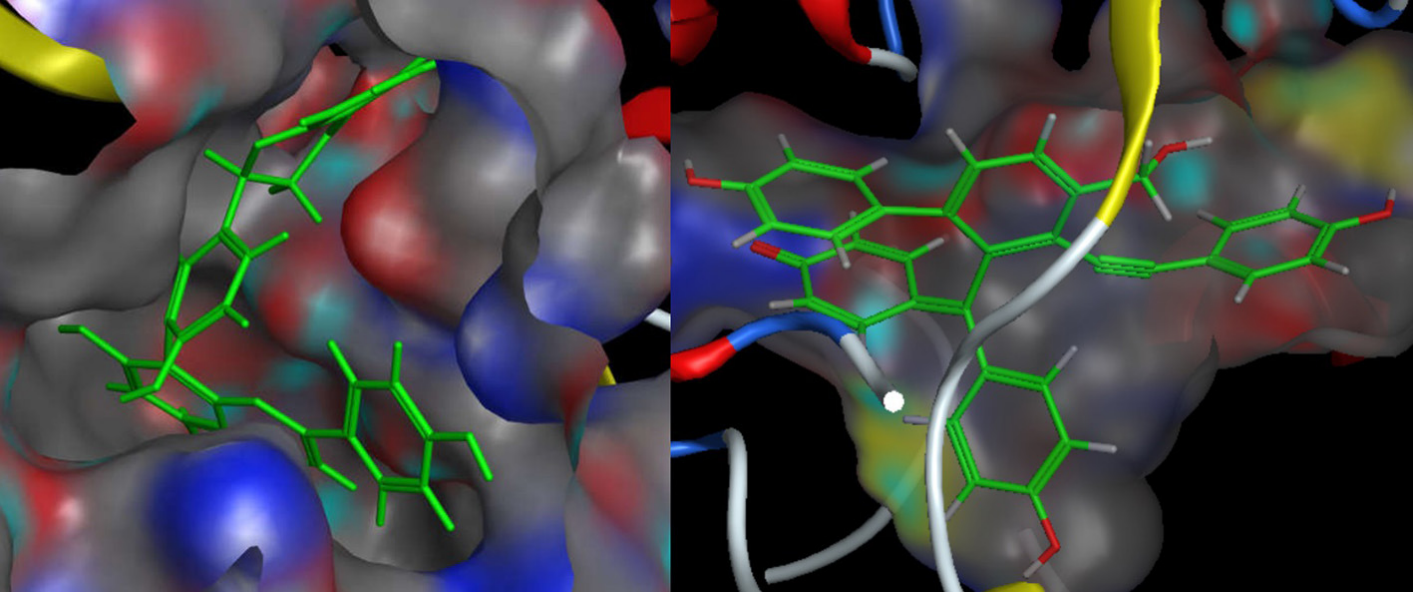
The docking results of S-8 bounding to BACE1 (**left**, PDB ID: 1TQF) and MAO-B (**right**, PDB ID: 2V5Z). The structure of S-8 is rendered green and the docking pocket surface was adjected to a suitable transparency.

**Figure 5:**
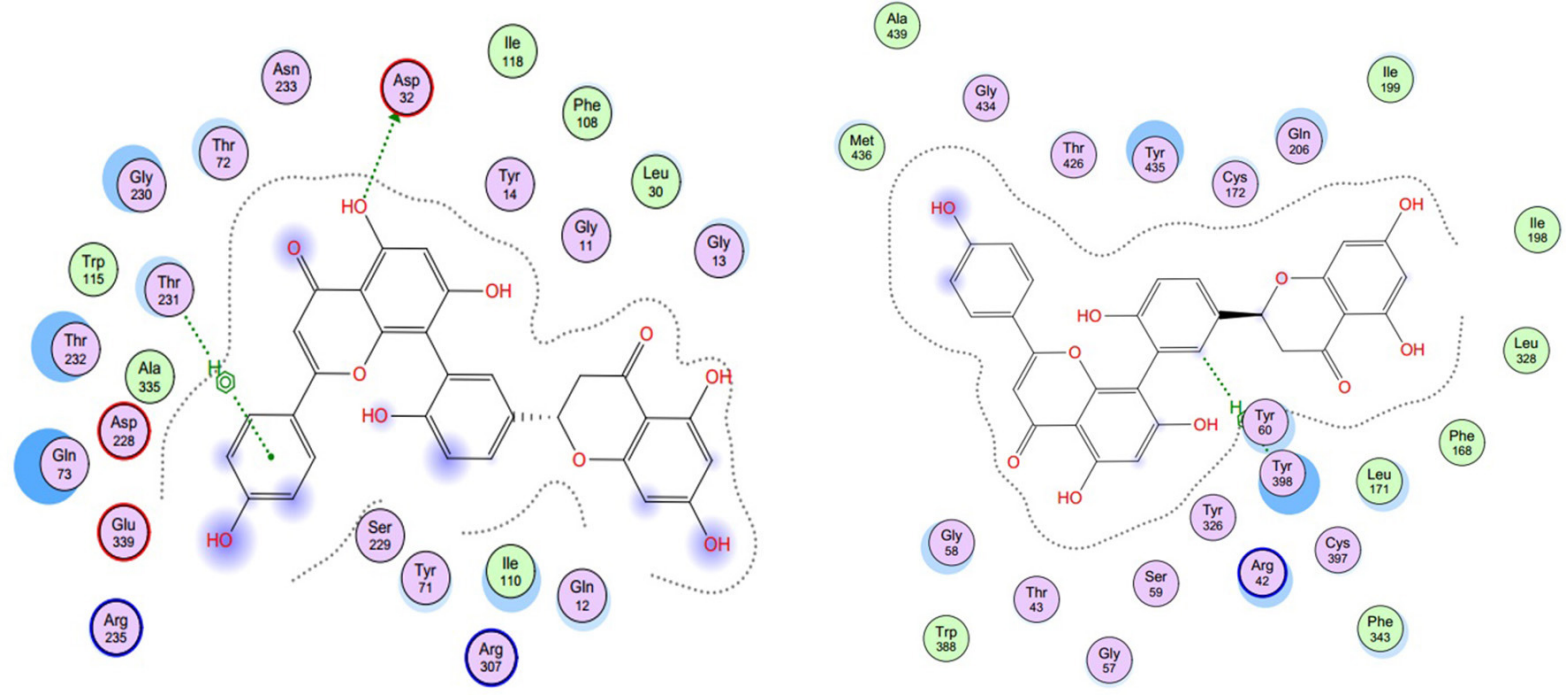
The ligand interaction diagram of S-8 bounding to BACE1 (**left**, PDB ID: 1TQF) and MAO-B (**right**, PDB ID: 2V5Z). It is a 2D diagram of the original ligand and a schematic representation of the binding site residues, with the important interactions between ligand and binding site shown. For BACE1, the main binding force is the hydrogen bond force and pi-bond force with ASP (232A) and THR (231A); for MAO-B, the main binding force is the hydrogen bond force and pi-bond force with CYS (397A) and GLY (13A).

As described before, for each structural conformation of S-5, S-8, S-12, and S-13, a score value was obtained to evaluate the binding affinity between this active compound and each target protein (BACE1 and MAO-B). Generally, a lower score is better. Therefore, the optimal conformation can be decided from a series of generated conformations for each compound according to their score values. Combining the result of ligand interaction, four active compounds and their docking results were listed in Table 4. From the table, we can see that four compounds indeed all have some degree of interaction with BACE1 and MAO-B compared with their original ligands. For BACE1, the most active molecule is S-8 for which the score value is −32.7 and the main binding force is the hydrogen bond force and pi-bond force with ASP (232A) and THR (231A). As to the rest three molecules, the mainly binding force are also the hydrogen bond and pi-bond force with different residues. For MAO-B, the most active molecule is S-5 for which the score value is −44.0 and the main binding force is the hydrogen bond force and pi-bond force with CYS (397A) and GLY (13A). In summary, the molecular docking results were consistent with the results of aforementioned inhibitory experiments that S-8 has the strongest inhibition activity for BACE1 and S-5/S-8 performs better than S-12/S-13 in the inhibition of MAO-B. Therefore, as the conclusion obtained from above *in intro* inhibitory test, S-5, S-8, S-12, S-13 all have significant dual BACE1/MAO-B inhibitory activities and S-8 promises to be the most potent against BACE1 and MAO-B. The docking results and corresponding 2D ligand interaction diagram of S-8 bound to BACE1 and MAO-B can be seen in Figures 4, 5. The detailed information of all conformations and docking results for other three compounds can be seen in the Supporting Information (Supplementary Material).

**Table 4:** Four active compounds and their docking results.

**Compound**	**BACE1**			**MAO-B**		
	**Score**	**Binding residues**	**Binding force**	**Score**	**Binding residues**	**Binding force**
S-5	−29.7	THR (232A); GLN (12A);THR (232A)	H-acceptor; pi-H; pi-H	−44.0	CYS (397A); GLY (13A)	H-donor; pi-H
S-8	−32.7	ASP (32A); THR (231A)	H-donor; pi-H	−38.4	TYR (398A)	H-pi
S-12	−27.8	THR (231A)	pi-H	−35.4	TRP (388A)	H-pi
S-13	−28.4	GLN (73A); ARG (307A)	H-donor; H-acceptor	−34.1	TRP (388A)	H-pi
Ligand	−35.8	–	–	−51.7	–	–

## Conclusion

In this study, we explored that the TE extracted from *Selaginella* plants has a remarkable improvement on learning and memory function for AD mice by Morris water maze test. And then, we preliminarily screened our home-database consisting of flavonoids compounds by multi-target SAR models *in silico*. After that, the *in vitro* enzyme activity inhibitory test was applied to evaluate 13 compounds that were considered to be active by multi-target SAR models and finally 4 compounds (S-8, S-5, S-13, and S-12) were found to have significant inhibitory activities on both BACE1 and MAO-B. Among them, S-8 has been proved to be the most potent ingredient against BACE1 and MAO-B with IC_50_ values of 0.745 and 3.619 μM, respectively. What's more, the molecular docking experiment was applied to verify the prediction results and to find the binding position and binding strength between the active ingredient and AD-related proteins. All in all, after these study and validation processes, we explored a new strategy to improve the efficiency of screening the active ingredients based on trace amount of natural product and numbers of targets and finally obtained some multi-targets potential compounds for the development of novel drugs for AD treatment.

## Author contributions

YD, NW, DC, and GT designed this study. YD and NW wrote and revised the manuscript. ZZ helped in preparing figures and tables. LZ, AC, and KX helped in giving suggestions to improve the manuscript. All authors read and approved the final manuscript.

### Conflict of interest statement

The authors declare that the research was conducted in the absence of any commercial or financial relationships that could be construed as a potential conflict of interest.

## Footnotes

^1^http://www.bindingdb.org/bind/index.jsp

## Supplementary material

Supporting Information SI1 lists 19 AD-related proteins and their detail information, the information including their gene names, uniport ID, protein name, and the number of samples for modeling process. Supporting Information SI2 lists the structural information of 18 compounds selected by multi-target SAR models. Supporting Information SI3 shows the detailed information of all conformations and docking results for S-5, S-12, and S-13.
