# Supplementary figures and images for: Multi-Target Screening and Experimental Validation of Natural Products from Selaginella Plants against Alzheimer's Disease
Source: Front Pharmacol. 2017 Aug 25;8:539. doi: 10.3389/fphar.2017.00539 (PMC5574911; doi:10.3389/fphar.2017.00539)

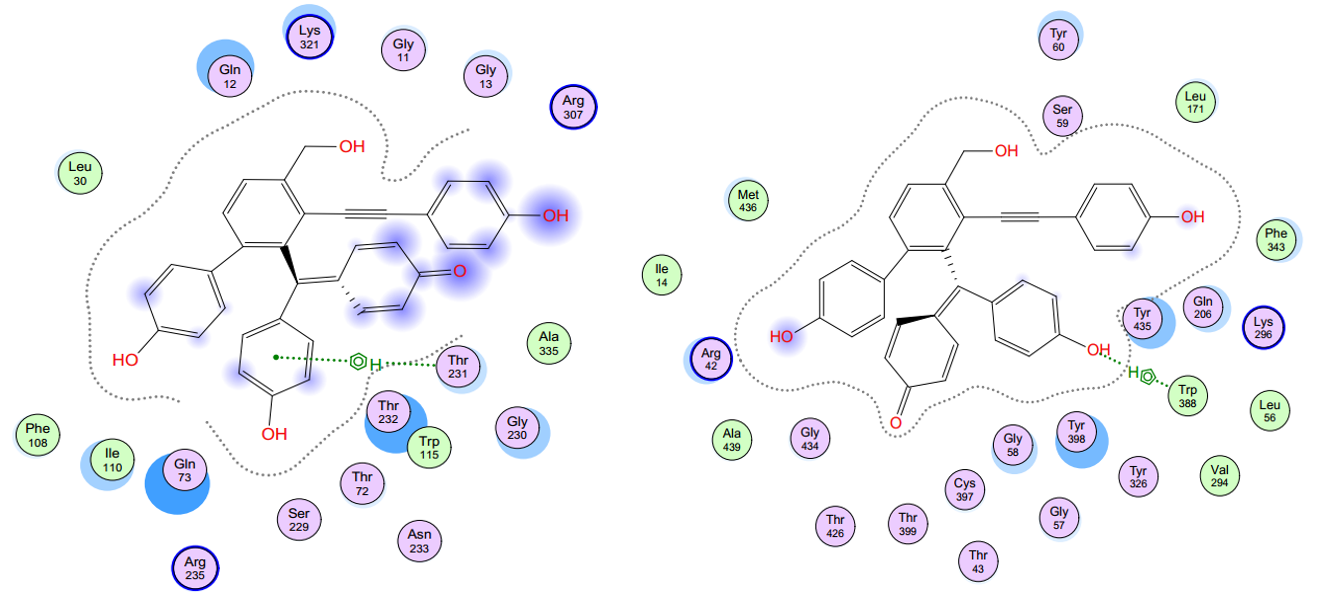

Supplement: Supplementary file 1 [file DataSheet1.ZIP › supporting information/SI-3-Molecular dockIng/S-12-inter.png]

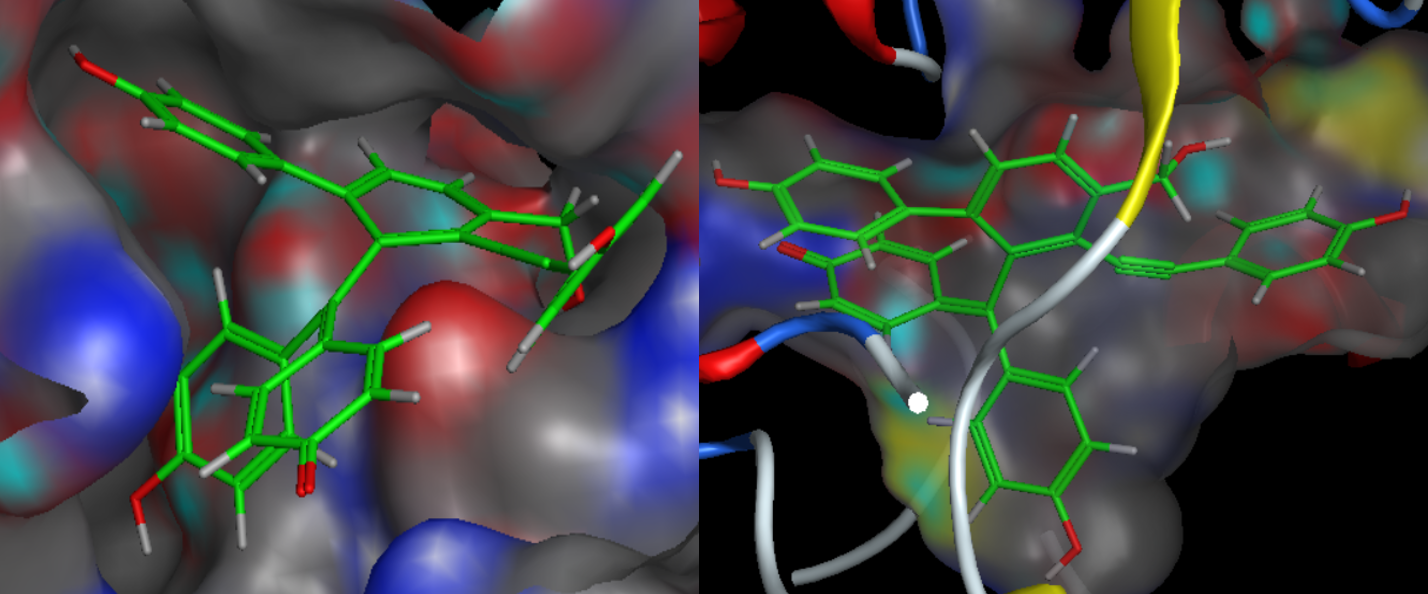

Supplement: Supplementary file 1 [file DataSheet1.ZIP › supporting information/SI-3-Molecular dockIng/S-12.png]

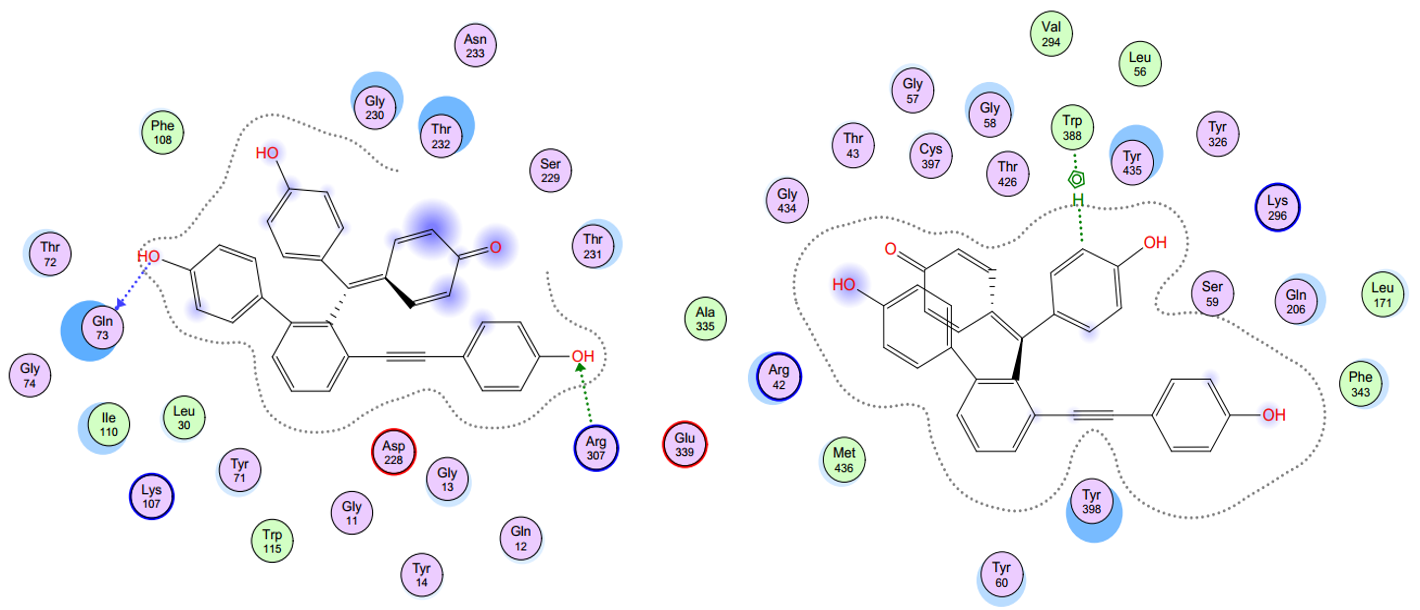

Supplement: Supplementary file 1 [file DataSheet1.ZIP › supporting information/SI-3-Molecular dockIng/S-13-inter.png]

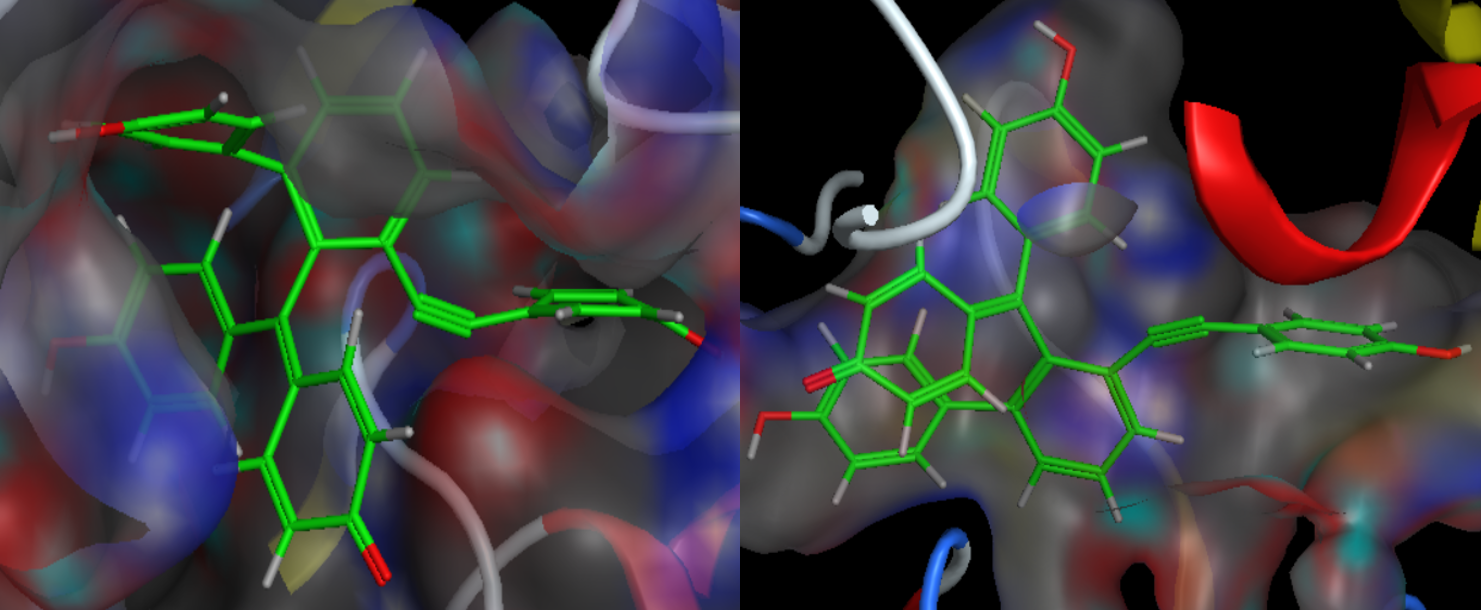

Supplement: Supplementary file 1 [file DataSheet1.ZIP › supporting information/SI-3-Molecular dockIng/S-13.png]

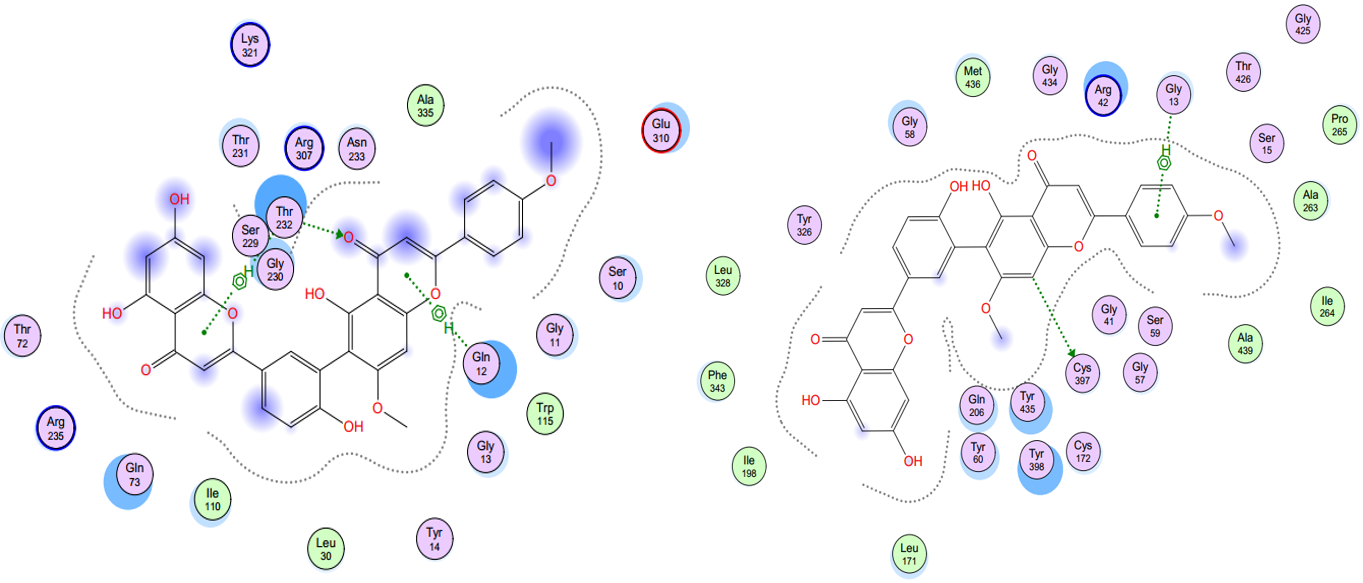

Supplement: Supplementary file 1 [file DataSheet1.ZIP › supporting information/SI-3-Molecular dockIng/S-5-inter.png]

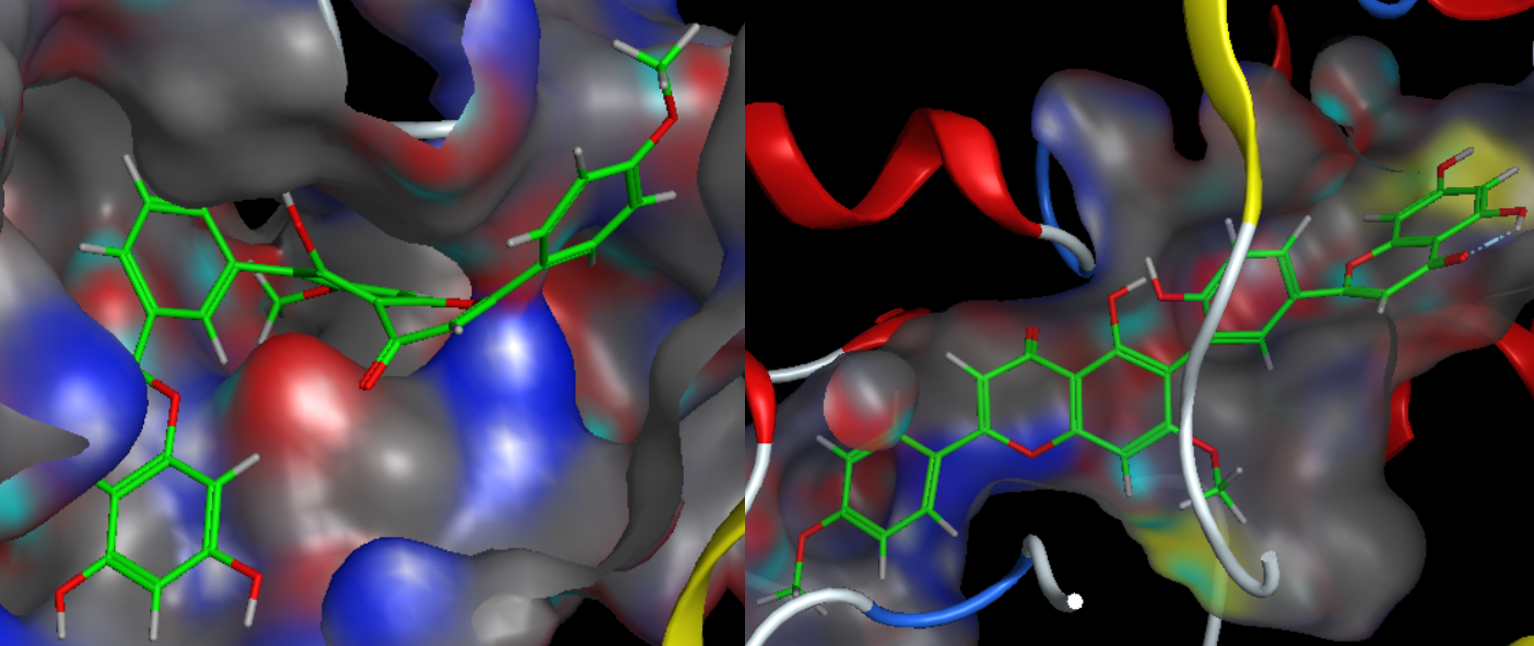

Supplement: Supplementary file 1 [file DataSheet1.ZIP › supporting information/SI-3-Molecular dockIng/S-5.png]
